# The immediate effectiveness of a multi-channeled oral irrigator in reducing dental plaque and oral malodor: A preliminary single-blind clinical trial

**DOI:** 10.1097/MD.0000000000046185

**Published:** 2026-05-12

**Authors:** Myong-Hwan Karm, Chan Gyu Kim, Jae-Young Lee, Hyun-Jae Cho, Hyun Jeong Kim

**Affiliations:** a Department of Dental Anesthesiology, School of Dentistry and Dental Research Institute, Seoul National University, Seoul, Republic of Korea; b Department of Artificial Intelligence Convergence, Chonnam National University, Gwangju, Republic of Korea; c Department of Dental Hygiene, College of Health Science, Dankook University, Chungcheongnam-do, Republic of Korea; d Department of Preventive Dentistry and Public Oral Health, School of Dentistry and Dental Research Institute, Seoul National University, Seoul, Republic of Korea.

**Keywords:** dental plaque, halitosis, oral health, oral microbiome, patient hygiene performance index, periodontal disease, preventive dentistry, therapeutic irrigation, volatile sulfur compounds

## Abstract

**Background:**

This study evaluated the immediate effectiveness of a multi-channeled oral irrigator (MCOI) in removing dental plaque and reducing oral malodor.

**Methods:**

In 20 healthy volunteers, changes in dental plaque were assessed using the Patient Hygiene Performance index. Volatile sulfur compounds were measured before and immediately after a single use of the MCOI. Participants refrained from tooth brushing for 24 hours prior to the assessment. The device administered 1000 mL of water over a 1.5-minute cycle. Oral malodor was analyzed with gas chromatography focusing on methyl mercaptan (CH₃SH) and hydrogen sulfide. Statistical analysis was performed using the Wilcoxon signed-rank test and chi-square test.

**Results:**

After a cycle of MCOI use, the average and divisions of the tooth Patient Hygiene Performance index scores significantly decreased (*P* < .001), with an average reduction of 24.76% in initial dental plaque. Additionally, total volatile sulfur compounds and CH₃SH concentrations significantly decreased (*P* = .011) after oral cleaning. Although the mean hydrogen sulfide concentrations decreased after oral cleaning, this change was not statistically significant (*P* = .088).

**Conclusions:**

Tooth brushing typically removes approximately 42% to 60% of the initial dental plaque in healthy adults. A single cycle of MCOI use reduced plaque by approximately 25% and significantly lowered CH₃SH, a compound associated with periodontal disease. These findings suggest that the MCOI may serve as a useful adjunct to tooth brushing, particularly for individuals with limited motor skills, and could help reduce the risk of plaque-related oral diseases.

**Key points**

- Single-cycle use of a multi-channeled oral irrigator (MCOI) removed approximately 25% of the initial dental plaque after a day without tooth brushing.
- The MCOI improves the effectiveness of tooth brushing in individuals with limited cognitive abilities or fine motor skills, including children, people with disabilities, and older people. Moreover, it effectively reduced the levels of volatile sulfur compounds, such as methyl mercaptan, which are linked to periodontal diseases.
- These findings suggest its significance as a complementary tool in daily oral care, improving dental plaque and malodor removal and potentially lowering the risk of dental plaque-related oral diseases, such as dental caries and periodontal diseases.

## 1. Introduction

Maintaining good oral health involves regular tooth brushing with proper techniques and frequency.^[1–3]^ Supplementary dental hygiene aids such as water flossers or single-channeled oral irrigators (SCOIs) like WATERPICK can also help prevent most dental diseases.^[4–7]^ Dental plaque begins to form approximately an hour after oral cleaning. It matures in approximately 72 hours.^[8]^ Factors such as dietary habits and the host immune system can influence plaque formation, and daily oral care habits play a crucial role in determining plaque buildup. Dental plaque is a significant risk factor for developing dental caries and periodontal diseases,^[9–11]^ which can ultimately result in tooth loss. Therefore, ensuring effective plaque removal during routine tooth brushing is essential for maintaining good oral health.

Appropriate tooth brushing techniques involve the effective removal of plaque from all tooth surfaces, including those in subgingival areas. However, this task can be challenging for individuals with limited cognitive abilities or fine motor skills, such as children, people with disabilities, and older adults. Consequently, these individuals may struggle to maintain good oral health independently. This highlights the importance of providing tailored oral care instructions and assistance to help them maintain optimal oral health.^[12]^

Although tooth brushing is the gold standard for plaque removal, it only removes approximately 42% to 60% of the initial dental plaque because of the challenge of achieving proper technique in healthy adults.^[1]^ Conversely, using a water flosser alongside manual tooth brushing is more effective in removing plaque from tooth surfaces than traditional string floss.^[5]^ This dual approach not only improves plaque removal but also reduces probing pocket depth, bleeding on probing (BOP), supragingival plaque index, and proinflammatory cytokine concentrations.^[13]^

Dental plaque and oral malodor result from inadequate oral hygiene, microbial activity, and host factors.^[14,15]^ Diagnosis commonly relies on plaque indices such as the Patient Hygiene Performance (PHP) index and gas chromatography for volatile sulfur compounds (VSCs).^[16,17]^ Besides conventional brushing and irrigators, other treatment modalities, including diode laser applications, have been shown to reduce bacterial load and improve esthetic outcomes.^[18,19]^

Halitosis or oral malodor, commonly known as bad breath, is not a frequent oral complaint but a distressing condition that may significantly affect the quality of life. In the developed world, 8% to 50% of people experience persistent recurrent episodes of halitosis. The most likely cause of halitosis is the accumulation of food debris and dental bacterial plaque on the teeth and tongue due to poor oral hygiene.^[14,15]^ Poor oral hygiene leads to microbial overgrowth, and intraoral surfaces are colonized by diverse bacterial species, especially anaerobes, which degrade substrates into VSCs^[20]^ and cause halitosis.

SCOIs have demonstrated greater efficacy in reducing gingival bleeding compared with traditional methods, such as toothpicks and manual flossing.^[21]^ However, a notable drawback of many water flossers is the requirement for users to position the nozzle precisely along the dentogingival or gum line of each tooth and to manually move it along this line. This task can be particularly challenging for individuals with cognitive limitations, dexterity issues, and motor-skill impairments.

**Figure 1.**
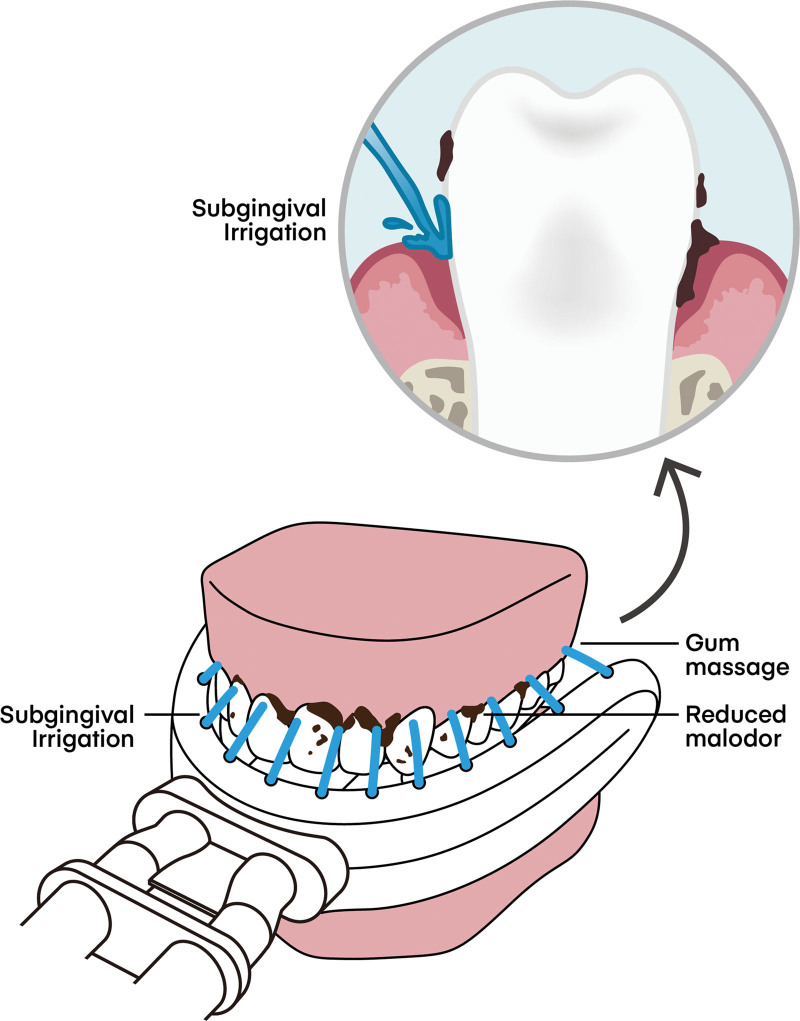
The multi-channeled oral irrigator enhances oral health by using pressurized, pulsatile water to remove food debris and dental plaque. This technology not only cleans surface areas but also reaches deep into gingival pockets, providing a more thorough cleanse than traditional brushing and flossing and effectively improving overall oral hygiene.

The multi-channeled oral irrigator (MCOI) provides a unique advantage by enabling comprehensive cleaning of all tooth surfaces with pressurized pulsatile water jets while simultaneously suctioning oral irrigated water to prevent pulmonary aspiration (Fig. 1). Clinical trials have shown significant improvements in plaque index, gingival index, BOP, and probing pocket depth when using the MCOI in addition to tooth brushing compared to tooth brushing alone.^[22]^ Furthermore, an investigation with healthy volunteers who underwent dental scaling 2 weeks prior and then used the MCOI for 3 consecutive days without concurrent tooth brushing resulted in significant changes in the plaque index, Sulcus Bleeding Index, and BOP as oral health indicators of periodontal diseases and a significant beneficial shift in the oral microbiome.^[23]^ Additionally, there was a significant reduction in the concentrations of VSCs after 2 weeks of using the MCOI compared to before its use.^[24]^ The pulsatile hydrokinetic pressure generated by the MCOI, along with its large volume of water, can effectively reduce dental plaque, including in subgingival areas.

The objective of this study was to quantify the immediate effectiveness of the MCOI in reducing dental plaque and VSCs in healthy volunteers who refrained from tooth brushing for 1 day prior to the assessment.

## 2. Materials and methods

This preliminary clinical trial was conducted at our institute in June 2019. It was an open-label, single-blind study approved by the Institutional Review Board of our institute (S-D20190013) and registered with the Clinical Research Information Service at https://cris.nih.go.kr (KCT0006515). The participants were provided with information about the study’s purpose and procedures and assured that declining to participate would not be disadvantageous to them. Written informed consent was obtained from all participants prior to enrollment. The study adhered to the principles outlined in the Declaration of Helsinki and Consolidated Standards of Reporting Trials (2010).

For this study, we recruited 20 healthy adult volunteers who met the following criteria: the presence of at least 16 natural teeth and no prosthetic restorations extending beyond the incisal edges or occlusal surfaces (e.g., laminates or crowns). Individuals with advanced periodontal disease, characterized by probing depths exceeding 4 mm, those using fixed orthodontic appliances, and current regular smokers were excluded.

**Figure 2.**
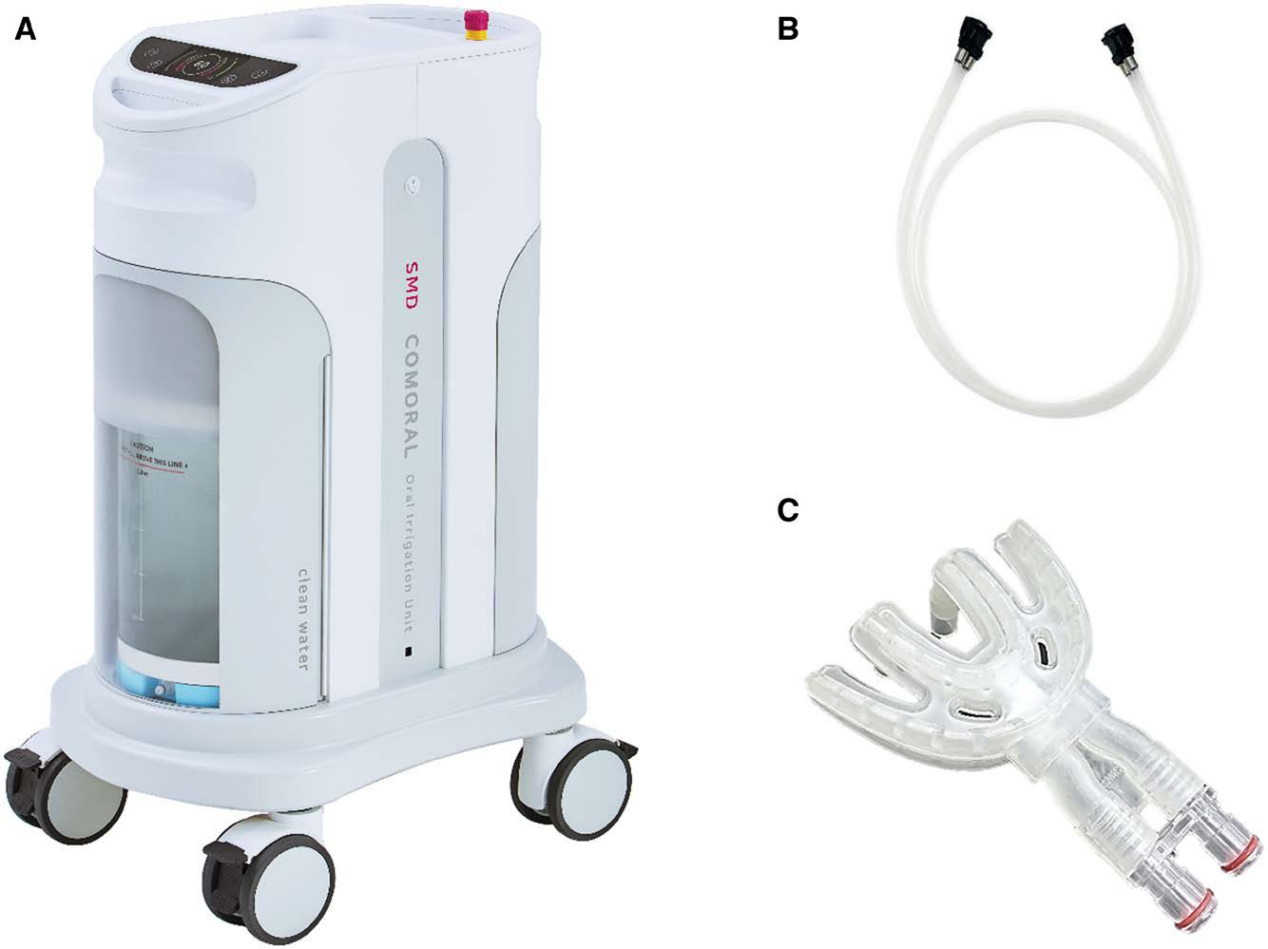
The institutional design of the multi-channeled oral irrigator (MCOI) used in this study. The MCOI comprises the following components: (A) water ejection and suctioning apparatus: 2 independent pumps facilitate the ejection of clean water from 20-L water tanks and simultaneously suction the oral irrigated water. (B) A 2-way connecting tube: 2 separate pathways, one for the influx of clean water and another for the efflux of used oral irrigated water, guarantee no mixing between the incoming and outgoing streams. (C) The mouthguard or WETERET, meaning scaling with water, has 60 nozzles allowing the injection of pressurized pulsatile water at the dentogingival junction and the suction of irrigated water to prevent pulmonary aspiration dividing clean and irrigated water within the mouthpiece.

We used an institutional COMORAL (SMDsolutions, Seoul, Korea) as the MCOI for the removal of dental plaque and reduction in oral malodor (Fig. 2). We administered 1000 mL of water over 1.5 minutes using the MCOI. To ensure standardized conditions for the measurements, participants were instructed to abstain from tooth brushing, gargling, and the consumption of gum and other oral hygiene products for 24 hours period prior to measurement. A water ejection and suctioning apparatus were equipped with pumps and 20-L water tanks. To assess the immediate efficacy of the MCOI in reducing dental plaque and halitosis, we measured the PHP index for assessing dental plaque^[21]^ and VSCs before and after using the MCOI following 24 hours of abstention from tooth brushing.

To visualize dental plaque for PHP index measurement, we applied a plaque-disclosing solution (Trace Solution, Young, Missouri) to all tooth surfaces using a cotton pellet, and then the solution was washed, and the tooth was dried. Images of dental surfaces were captured using a digital camera (D5600; Nikon, Tokyo, Japan). The PHP index was measured on a specific tooth surface. Each tooth surface was divided into 3 equal parts, namely the mesial, distal, and central regions. The central part was further divided into 3 equal parts, namely the gingival, central, and occlusal regions, resulting in a total of 5 parts. Each part was scored based on whether it was colored (1 point) or not (0 points) after applying the plaque-disclosing solution, indicating the presence or absence of dental plaque. The scores of the 5 divisions for each tooth were then combined to obtain the tooth score, and the scores for all 6 teeth were combined. The final score was calculated by dividing the sum by 6, the total number of teeth examined. The final score represented the participant’s PHP index.^[16]^

Halitosis in the study participants was evaluated by measuring the concentration of VSCs in the morning following specific guidelines, which included refraining from eating after brushing their teeth in the evening until the next morning. Gas chromatography (Twin Breasor II, iSenLab Inc., Seoul, Korea) was used to assess oral malodor by assessing the concentrations of methyl mercaptan (CH_3_SH) and hydrogen sulfide (H_2_S). The participants held a dedicated straw in their mouth and inhaled it for 10 seconds after nasal respiration for 50 seconds. The analysis was conducted 150 seconds after inhalation, and the results are presented as graphs and values. According to the manufacturer’s user guide, oral malodor was determined if the values of CH_3_SH and H_2_S were above a certain threshold.^[25]^

Data were analyzed using IBM SPSS Statistics (version 25.0; IBM Corp., Armonk). Participants were categorized into 2 groups: an oral malodor group (CH_3_SH ≥ 26 ppb or H_2_S ≥ 112) and a non-oral malodor group (VSCs = 0) based on the concentration of VSCs. The effects of MCOI were assessed using the Wilcoxon signed-rank test to compare the mean PHP index scores and the chi-square test to compare changes in VSCs (*P* < .05).

## 3. Results

Twenty healthy participants were recruited for the preliminary study. A detailed description of this process is provided in Table 1.

**Table 1:** Demographics of participants.

Variables	
Age (yr)	
Mean	27.0
SD	3.6
Range	24–37
Gender	
Male	10 (50%)
Female	10 (50%)

SD = standard deviation.

**Figure 3.**
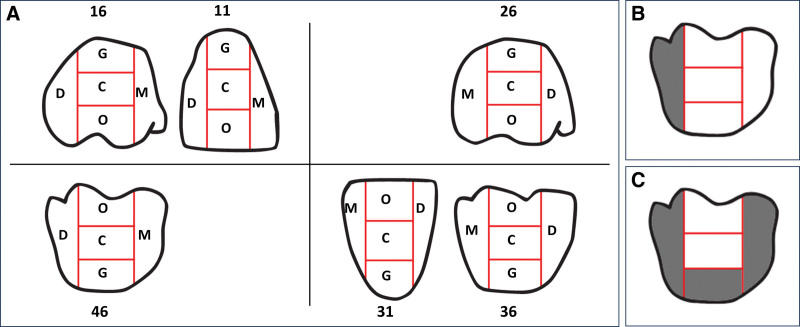
The divisions of a tooth used in scoring for the patient hygiene performance (PHP) index scores, (A) 5 divisions at the observed 6 teeth, (B) when the PHP index score of 1, and (C) when the PHP index score of 3. C = central; D = distal; G = gingival; M = mesial; O = occlusal.

**Figure 4.**
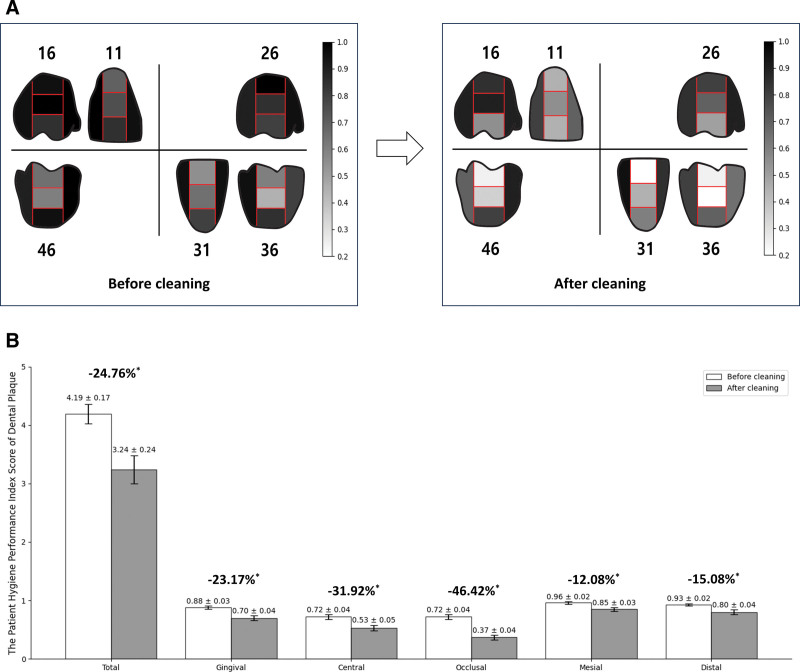
The mean changes in the patient hygiene performance (PHP) index scores before and after oral cleaning. (A) The visually observed decreases in the mean PHP index scores across all divisions of the measured teeth (#16, #11, #26, #36, #31, and #46) before and after oral cleaning. The different shades of gray represent the mean PHP index at each division of the tooth between white (0) and black (1). (B) The significant decreases in the mean PHP index scores before and after oral cleaning indicate significant improvements in plaque removal. The mean PHP index scores across all divisions of the measured 6 teeth suggest that the MCOI effectively removes dental plaque from the whole surface of the tooth. Furthermore, the average individual percentage change of the PHP index scores was −24.76%, indicating a substantial reduction in dental plaque after oral cleaning with the MCOI (*P < .001).

A statistically significant decrease in the PHP index was observed after a single use of the MCOI. The mean PHP index score significantly decreased from 4.19 ± 0.76 to 3.24 ± 1.07 (*P* < .001), with a percent change of −24.76% from baseline (Fig. 3). Among the 5 areas measured in 6 teeth (#16, #11, #26, #36, #31, and #46), greater plaque distribution was observed before cleaning, particularly in the mesial and distal areas of each tooth. However, after oral cleaning with the MCOI, there was a significant decrease in the amount of dental plaque attached to all 6 teeth, particularly in the 3 middle divisions of each tooth (*P* < .001; Fig. 4).

Regarding the effect of the MCOI in reducing VSCs, the mean ± standard error of VSCs such as CH_3_SH and H_2_S were measured in parts per billion by volume (ppb, nmol/mL). The concentration of VSCs can vary depending on the measurement device used, dietary habits, oral hygiene practices, and overall health status, even within the same individual. Among the 20 participants, 6 showed no detectable VSCs in their initial samples, which is similar to that observed in a previous study.^[17]^ The concentration values of VSCs were below the cognitive threshold, and the concentrations at which the human nose detected malodor, as proposed by the manufacturer, were 26 ppb and 112 ppb for CH_3_SH and H_2_S, respectively. Participants with thresholds of H_2_S < 112 ppb and CH_3_SH < 26 ppb were classified into the non-malodorous group (n = 12), and those with more than these thresholds were classified into the oral malodorous group (n = 8; Table 2).

**Table 2:** Changes in concentrations of volatile sulfur compounds (VSCs) in participants with oral malodor (unit: ppb, %change).

	Methyl mercaptan (CH_3_SH)			Hydrogen sulfide (H_2_S)		
	Before cleaning	After cleaning	% change	Before cleaning	After cleaning	% change
1	52	10	19.23	280	306	109.29
2	246	108	43.90	1030	590	57.28
3	52	25	48.08	127	474	373.23
4	8	0	0.00	132	240	181.82
5	26	24	92.31	143	494	345.45
6	2	0	0.00	198	106	53.54
7	48	10	20.83	206	122	59.22
8	51	19	37.25	258	399	154.65
Average†	60.63 ± 27.44	24.5 ± 12.41*	40.41	296.75 ± 106.66	341.38 ± 62.83	115.04
*P*-value	.012*			.484		

% changes = change from before cleaning.

† Mean ± standard error.

* *P* < .05.

**Figure 5.**
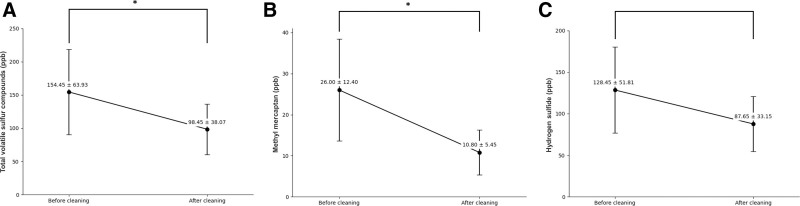
The mean changes in concentrations of total volatile sulfur compounds (VSCs), including methyl mercaptan (CH_3_SH) and hydrogen sulfide (H_2_S), before and after oral cleaning. (A) Total VSCs showed a significant decrease after oral cleaning compared to before cleaning (*P* = .034). (B) After oral cleaning, there was a significant decrease in CH_3_SH concentrations related to periodontal disease compared to before cleaning (*P* = .011). (C) Mean value of H_2_S concentrations decreased after oral cleaning compared to before cleaning. Nonetheless, the differences observed before and after cleaning were not statistically significant (*P* = .088). Each line represents mean ± SE (*P < .05).

Regarding the oral cleaning effect of a single cycle of the MCOI use on reducing VSCs, the mean concentrations of total VSCs and CH_3_SH significantly decreased from 154.45 ± 63.93 and 26.00 ± 12.40 ppb before cleaning to 98.45 ± 38.07 (*P* = .034) and 10.80 ± 5.45 ppb (*P* = .011) after cleaning, respectively (Fig. 5A and B). Similarly, average concentrations of H_2_S decreased from an initial measurement of 128.45 ± 51.81 ppb before cleaning to 87.65 ± 33.15 ppb after cleaning (Fig. 5C). However, this decrease was not statistically significant (*P* = .088).

## 4. Discussion

In this study, a single cycle of the MCOI significantly decreased dental plaque and oral malodor.

The evaluation of dental plaque is essential for assessing oral health. In this study, we used the PHP index to quantify dental plaque adhering to 5 areas of the tooth.^[26]^ The PHP index is known for its simplicity and robust inter-examiner correlations, making it suitable for use in research. Specifically, previous studies have shown correlations of 0.85 and 0.87 with the dye tablet method,^[16]^ affirming its suitability for use in research investigations. There was a remarkable reduction of 24.76% in the PHP index score following MCOI use compared with the measurements taken just before its use. This reduction was noted after 1 day of tooth brushing in healthy individuals, allowing for the accumulation of dental plaque, which typically begins within minutes and reaches full development within 3 days of tooth brushing.^[8]^ The decision to change the clinical protocol from a 3-day to 1-day absence without tooth brushing was made following the recommendations of the Institutional Review Board.

The concentrations of VSCs, specifically CH_3_SH and H_2_S, were measured before and immediately after a 1.5-minute cycle of MCOI use in this study. These VSCs are major contributors to oral malodor and are produced as end products of the proteolytic processes by oral microorganisms.^[27]^ The production of these VSCs is linked to the bacterial metabolism of sulfur-containing amino acids, such as cysteine and methionine.^[28]^ Various bacterial populations of the oral microbiota produce high concentrations of CH_3_SH or H_2_S in the oral cavity.^[29]^ Consequently, CH_3_SH and H_2_S are predominantly associated with oral malodor, whereas dimethyl sulfide, another VSC, may contribute to extra-oral or blood-borne malodor through uncertain metabolic pathways.^[30]^ Therefore, the total concentration of VSCs was calculated by summing the CH_3_SH and H_2_S concentrations in this study.

Gram-negative anaerobic bacteria are the predominant producers of CH_3_SH. Bacterial strains that produce large amounts of CH_3_SH (≥70 nmol/mg of protein/hour) from 1 mM of L-methionine were identified in the genera *Fusobacterium*, *Bacteroides*, *Porphyromonas*, and *Eubacterium*. Particularly, chronic peritonitis-related bacteria such as *Porphyromonas gingivalis*, *Fusobacterium nucleatum*, and *Treponema denticola* produce CH_3_SH from l-methionine through the action of L-methionine-α-deamino-γ-mercaptomethane lyase.^[31–33]^

After oral cleaning with the MCOI, we observed a significant decrease in the CH_3_SH concentration with baseline. Oral malodor can be clinically classified into 2 physiological and pathological types: genuine halitosis, pseudo-halitosis, and halitophobia.^[34]^ There are 2 types of genuine halitosis: physiological and pathological. Physiological halitosis, often experienced upon awakening, is commonly associated with H_2_S-related malodors. Conversely, pathological halitosis is primarily caused by the accumulation of food debris and dental bacterial plaques on the teeth and tongue due to poor oral hygiene. This accumulation leads to the gingival and periodontal inflammation associated with CH_3_SH production.^[14,34]^

Participants with preexisting halitosis showed reduced concentrations of CH_3_SH and H_2_S after oral cleaning (Table 2). In a 28-day clinical trial comparing tooth brushing and MCOI usage with tooth brushing alone, significant differences were observed in the plaque index, gingival index, BOP, and probing pocket depth.^[22]^ Additionally, another study involving healthy volunteers who underwent dental scaling followed by 3 days of MCOI usage without tooth brushing showed improved oral hygiene, including reduced BOP and favorable shifts in the oral microbiome.^[23]^ Furthermore, using the MCOI for 4 weeks resulted in decreased concentrations of VSCs compared with the baseline measurements obtained via gas chromatography. Notably, consistent standard deviations of approximately 8 ppb were observed after 2 weeks of MCOI use compared with a baseline of 22.3 ppb in 17 volunteers.^[24]^ These findings highlight the effectiveness of the MCOI in maintaining oral health and preventing halitosis.

The present findings are biologically plausible given that pulsatile hydrokinetic irrigation enhances removal of plaque biofilm and food debris, including in subgingival areas, thereby lowering bacterial substrates responsible for volatile sulfur compound production. Our observation of a 24.8% plaque reduction and significant decrease in CH₃SH levels is consistent with previous reports demonstrating the efficacy of MCOIs. For example, Kim et al showed in a randomized controlled trial that MCOI use in addition to tooth brushing significantly improved plaque and gingival indices compared with tooth brushing alone.^[22]^ Similarly, a 4-week trial reported by Kim and Kim demonstrated reductions in oral malodor and plaque indices after regular use of a new concept oral irrigator.^[24]^ These studies support our results and further indicate that MCOI use may provide both immediate and sustained benefits. In summary, the present trial provides short-term evidence supporting the use of oral irrigators as an effective adjunctive tool for improving oral hygiene and controlling halitosis.

In 2001, the American Academy of Periodontology confirmed that using water flossers could reduce gingival inflammation beyond that achieved by tooth brushing alone.^[4]^ The original SCOI, such as WATERPICK, uses compression and decompression phases within the gum tissue to remove supragingival plaque and flush out subgingival bacteria and debris.^[12]^ This process creates 2 hydrokinetic activity zones: an impact zone at the gingival margin and a flushing zone below the dentogingival junction. Water effectively reduced bacteria without long-term side effects, and the participants found the experience pleasant, resulting in a feeling of enhanced oral cleanliness.

A notable feature of MCOIs compared with SCOIs is their unique mouthpiece. It has 60 water-ejecting nozzles that efficiently and simultaneously release pressurized water, ensuring thorough coverage of the entire mouth. This design enhances the efficacy of oral irrigation compared with that of water flossers. In addition, it incorporates a synchronized mechanism in which water ejection and suction occur through the same mouthpiece. This prevents the potential aspiration of the orally irrigated water-containing bacteria into the lungs, thereby guaranteeing safer and more effective oral irrigation. Recent meta-analysis highlighted the significant effects of chronic periodontitis on the clinical course of coronavirus (SARS-CoV-2) disease 2019 with severe acute respiratory syndrome. Chronic periodontitis has been associated with a fourfold increase in the likelihood of hospitalization among affected patients, and maintaining good oral hygiene is crucial for public health.^[35]^

This study had several limitations. First, the sample size was small, which may have limited the generalizability of the findings. Second, measuring VSCs presents technological challenges, and controlling confounding factors can be challenging. However, CH₃SH is considered a reliable indicator. Third, there are various methods for measuring dental plaque, and the choice of method can affect the results. Further research and clinical trials with larger sample sizes and alternative measurement approaches are needed to evaluate the long-term effects and broader implications of the MCOI for oral health maintenance and disease prevention.

In conclusion, single-cycle use of the MCOI resulted in a 24.76% reduction in initial dental plaque levels, along with a decrease in CH_3_SH levels. These findings suggest that the MCOI may serve as a useful adjunct to tooth brushing, particularly for individuals with limited motor skills, and could help reduce the risk of plaque-related oral diseases. Further research is warranted to explore long-term efficacy and broader effects on oral health.

## Acknowledgments

We express our sincere appreciation to Seon-Jip Kim of the Department of Preventive Dentistry and Public Oral Health, School of Dentistry and Dental Research Institute, Seoul National University, and Ji Yeon Choo of SMDsolutions Co., Ltd. for their contributions to this clinical trial. The authors would like to thank the Dental Research Institute of Seoul National University for the English language review.

## Author contributions

**Conceptualization**: Hyun-Jae Cho, Hyun Jeong Kim.

**Data curation**: Jae-Young Lee.

**Formal analysis**: Chan Gyu Kim.

**Funding acquisition**: Hyun Jeong Kim.

**Investigation**: Jae-Young Lee.

**Methodology**: Hyun-Jae Cho.

**Project administration**: Hyun-Jae Cho.

**Resources**: Jae-Young Lee.

**Supervision**: Hyun-Jae Cho.

**Validation**: Hyun-Jae Cho.

**Visualization**: Myong-Hwan Karm, Chan Gyu Kim, Hyun Jeong Kim.

**Writing – original draft**: Myong-Hwan Karm, Chan Gyu Kim, Hyun Jeong Kim.

**Writing – review & editing**: Myong-Hwan Karm, Hyun Jeong Kim.
